# Anatomical and Electrophysiological Characteristics of Dual-Loop Re-Entry in Atypical Atrial Flutter: Implications for Mapping and Catheter Ablation

**DOI:** 10.3390/jcm13102847

**Published:** 2024-05-12

**Authors:** Nicolas Johner, Mehdi Namdar, Dipen C. Shah

**Affiliations:** Cardiology Division, Geneva University Hospitals, Rue Gabrielle-Perret-Gentil 4, 1205 Geneva, Switzerland; nicolas.johner@hug.ch (N.J.); mehdi.namdar@hug.ch (M.N.)

**Keywords:** atrial flutter, atrial tachycardia, dual-loop, re-entry, macro-re-entry, catheter ablation, isthmus, mapping, substrate

## Abstract

**Background:** Atypical atrial flutter (AFL) can be challenging to ablate, especially when involving dual-loop re-entry. We sought to assess the electroanatomical characteristics of single- and dual-loop AFLs in patients undergoing catheter ablation. **Methods:** We analyzed 25 non-cavotricuspid isthmus-dependent macro-re-entrant AFL in 19 consecutive patients. Three-dimensional high-density activation mapping was performed, and active re-entry loops were confirmed by entrainment mapping. **Results:** Of 25 AFLs (24 left, 1 right atrial), 13 (52%) exhibited dual-loop re-entry. The most common circuits included, in 6/13 (46% of dual loops), a perimitral re-entry with a second loop around the right/left pulmonary veins (PV) and, in 6/13 (46%), involved a right PV ostium with a second loop around either a functional conduction block or another PV. Ablation at the common isthmus of dual-loop AFLs and at the critical isthmus of single-loop AFLs terminated the arrhythmia more frequently than ablation at a secondary isthmus of dual-loop AFLs (5/6 (83%) and 8/11 (73%) versus 1/8 (13%), respectively, *p* = 0.013). **Conclusions:** More than half of AFLs exhibited a dual-loop re-entrant mechanism. Most critical isthmuses were found at the mitral isthmus, the left atrial roof or right PV ostia. Ablation targeting the common isthmus resulted in a higher termination rate.

## 1. Introduction

Atypical atrial flutter (AFL) represents a heterogeneous set of complex arrhythmias defined as non-cavotricuspid isthmus (CTI)-dependent macro-re-entrant atrial tachycardia. The arrhythmogenic substrate generally consists of combination(s) of anatomical obstacles (valves and other orifices) and left or right atrial myocardial scars, the latter often resulting from catheter ablation [1,2,3] (e.g., atrial fibrillation [AF] ablation) or cardiac surgery [2,4,5], but may also occur without prior interventions in the setting of advanced atrial cardiomyopathy [6]. AFL is often difficult to manage medically and catheter ablation is an established treatment. Because AFL has highly variable anatomy, the selection of appropriate ablation targets relies on the individualized identification of critical isthmuses within the re-entry circuit. Three-dimensional (3D) high-density electroanatomical mapping allows delineation of the complete re-entry circuit and facilitates the identification of narrow slow-conducting critical isthmuses [7]. Despite modern tools, however, AFL can be challenging to map and ablate, owing in part to its sometimes complex and frequently variable anatomy, and long-term rates of freedom from arrhythmia after catheter ablation have been modest. In one of the largest studies on complex atrial tachycardias, Derval et al. reported recurrence in 45 of 101 (45%) patients after ablation of re-entrant atrial tachycardia during a follow-up of 13 ± 9 months [2]. Dual-loop re-entry (or figure-of-8 re-entry) can be defined by the presence of two synchronous active loops without delay compared to other loops and confirmed by entrainment mapping. Dual-loop AFLs pose specific challenges due to circuit complexity and the multiplicity of potential ablation targets. While dual-loop atrial macro-re-entry was first reported more than two decades ago [5], data remains scarce regarding its prevalence, electroanatomical characteristics and optimal ablation strategy. In the largest study on multiple-loop AFL ablation, Takigawa et al. reported recurrence of atrial tachycardia in 19 of 42 (45%) patients 1 year after ablation [1], highlighting the challenge that these arrhythmias represent.

We sought to assess the prevalence of dual-loop re-entry and to describe its electroanatomical characteristics in patients undergoing catheter ablation of AFL using high-density 3D electroanatomical mapping.

## 2. Methods

### 2.1. Design

We retrospectively studied 19 consecutive patients who underwent catheter ablation of AFL at our institution. AFL was defined as non-CTI-dependent atrial tachycardia with regular monomorphic atrial activity and a re-entrant mechanism confirmed by electrophysiology study. CTI-dependent atrial flutters were excluded from the analysis. Inclusion criteria were: (1) clinically-indicated catheter ablation of AFL and (2) availability of at least one complete electroanatomical map (see Section 2.2. Electrophysiology Study) confirming a non-CTI-dependent re-entrant mechanism. The exclusion criterion was the absence of any documented non-CTI-dependent re-entrant AFL at electrophysiology study. Written informed consent was obtained for the procedures and the study was approved by the institutional review board.

### 2.2. Electrophysiology Study

Antiarrhythmic drugs were discontinued 5 half-lives before ablation, except amiodarone, which was discontinued for >3–10 days. A 10-electrode mapping catheter (5 mm electrodes, 2–5–2 mm spacing, Abbott, Abbott Park, IL, USA) was placed in the coronary sinus (CS) with the catheter tip in the distal CS or great cardiac vein and all electrodes inside the CS. Another identical catheter was placed on the right atrium (RA) free wall with the catheter tip close to the lateral CTI. Left atrial (LA) access was routinely obtained up-front by transseptal puncture under fluoroscopic guidance. Entrainment mapping was performed and, in case of a post-pacing interval (PPI) to tachycardia cycle length (TCL) difference (PPI-TCL) ≥ 40 ms at the CTI and at ≥ 2 sites on the RA free wall, ruling out RA AFL, LA mapping was performed.

High-density 3D electroanatomical maps were acquired sequentially (Rhythmia mapping system, Boston Scientific, Marlborough, MA, USA) during ongoing arrhythmia using a 64-electrode basket catheter (IntellaMap Orion, Boston Scientific). A re-entrant mechanism was confirmed based on documentation of >90% of TCL with early-meets-late circular activation. Entrainment mapping was performed at several candidate sites to confirm active re-entry loops. Entrainment was obtained at a pacing cycle length 15–20 ms shorter than TCL at a 25 mA output from the distal bipole of the ablation catheter (TactiCath Quartz Contact Force Ablation Catheter, Abbott). Entrainment was defined based on conventional criteria and a PPI-TCL < 20 ms was considered indicative of a site within an active component of the re-entry circuit. Entrainment locations and the corresponding PPI measures were routinely annotated on the electroanatomical maps for subsequent analysis.

Radiofrequency ablation was performed by targeting the narrowest accessible critical isthmus, aiming for both arrhythmia termination and anatomically complete ablation across the targeted isthmus. On an individual basis, the potential impact of ablation lesions on sinus rhythm atrial activation was considered at operator’s discretion when selecting ablation targets (e.g., to avoid the risk of left atrial appendage isolation in case of extensive LA scarring). After flutter termination, lesions were assessed based on abolition of local electrograms or the presence of double potentials. When applicable, differential pacing or activation mapping in sinus or paced rhythm was performed to confirm conduction block across ablation lines. When applicable, in patients with a history of AF or pulmonary vein isolation, PVs were mapped in sinus rhythm and re-isolated in case of reconnection. Following ablation, arrhythmia inducibility was assessed by atrial burst pacing in 10 ms decrements from 300 to 200 ms and incremented back to 300 ms. In case of induced arrhythmia sustained for >5 min, mapping and ablation were pursued at operator’s discretion based on estimated clinical benefit. Procedure endpoints were arrhythmia termination, non-inducibility of sustained arrhythmias by atrial burst pacing, and, when applicable, conduction block confirmation across ablation lines and PV isolation.

Bipolar electrograms were filtered (band-pass 30–500 Hz) and digitally recorded at a sampling frequency of 1000 Hz (LabSystem Pro, Boston Scientific) along with surface ECG.

### 2.3. Electroanatomical Map Analysis

The leading tachycardia wavefront was identified from activation maps based on the tip of consecutive isochrones indicative of the shortest active re-entry loop. Multi-loop re-entry was defined by the presence of ≥2 synchronous active loops without delay compared to other leading wavefronts and confirmed by entrainment mapping (PPI-TCLs < 20 ms). Isthmuses were defined as relative narrowing < 30 mm along the path of the leading re-entry wavefront(s) and delimited on both sides by the presence of anatomical obstacles (e.g., valve annulus or thoracic veins), electrical scars (bipolar electrogram voltage < 0.05 mV) or lines of conduction block (identified based on activation mapping). In case of dual-loop re-entry, a common isthmus was defined as an isthmus < 30 mm located along the common segment between both re-entry loops, while a secondary isthmus was defined as an isthmus < 30 mm at any other location along the active re-entry wavefronts. Isthmus width was measured using Rhythmia’s distance tool. Isthmus conduction velocity was calculated as the time interval between wavefront entry and exit to and from the isthmus divided by isthmus length. Entry and exit locations were identified as the sites with an abrupt change in width and/or in the direction of wavefront propagation, representative of the extremities of a protected channel. In the absence of a protected channel, i.e., isthmuses < 5 mm in length (localized narrowing), isthmus conduction velocity was measured by selecting two representative sites around the center of the isthmus, with an axis parallel to wavefront propagation and a distance of 5–15 mm (depending on map density at that site). Bipolar electrogram voltage was measured from representative electrograms (corresponding to the most common amplitude) within the isthmus. When available, PPI measures within the isthmus were verified offline (to exclude incorrect annotation or TCL variability) and PPI-TCL was calculated.

### 2.4. Acute Ablation Outcome

The effect of ablation at the targeted isthmus was defined based on the following categories: (1) AFL termination to sinus rhythm, (2) sustained increase in TCL by >15 ms, (3) manifest modification of the atrial activation sequence, and (4) absence of manifest effect on the arrhythmia.

### 2.5. Statistical Analysis

Unless otherwise specified, continuous variables are expressed as mean (±standard deviation) if normally distributed and as median (interquartile range) if not. Categorical variables are expressed as number (percentage). The distribution of continuous variables was assessed by visual examination of histograms and the Kolmogoroff-Smirnov test. Normally distributed continuous variables were compared using paired and unpaired two-tailed t-test (Welch approximation) as appropriate. Continuous variables with non-normal distribution were compared using the Mann-Whitney U-test, or the Kruskal-Wallis rank test for comparisons involving ≥ 3 groups. For categorical variables, Pearson’s Chi-squared test was used when expected frequencies were >5 across all categories and the Fischer exact test was used in case of expected frequencies ≤ 5. Statistical significance was defined at a bilateral alpha < 0.05.

## 3. Results

### 3.1. Clinical Characteristics

Baseline clinical characteristics are summarized in Table 1. Nineteen patients (age 66 ± 15 years, 79% male) who underwent 20 catheter ablation procedures were included and a total of 25 AFLs were analyzed, for which complete high-density electroanatomical activation maps were acquired and entrainment mapping at several candidate sites was performed. A presumed etiology of AFL was identified in 17 (89%) patients: 14 (74%) patients had undergone previous AF ablation (of which 13 had a history of prior AF ablation and 1 exhibited AFL during de novo AF ablation), 2 (11%) patients had undergone previous cardiac surgery (mitral and aortic valve replacement along with Maze procedure in one, and mitral valve repair along with Maze, patent foramen ovale closure and coronary artery bypass graft in one) and 1 (5%) patient had a history of bipulmonary transplant (and exhibited re-entry around the right pulmonary veins [PVs] suture). AFL had no clear etiology in 2 (11%) patients: one patient only had a history of medically managed moderate-to-severe (grade 3/4) tricuspid regurgitation (but exhibited left atrial flutter) and one patient had no significant past medical history except for arterial hypertension and no evidence of structural heart disease (except for tachycardiomyopathy, which recovered fully after catheter ablation of AFL).

### 3.2. Electroanatomical Mapping

Out of 25 AFL, 13 (52%) exhibited a dual-loop re-entrant mechanism and 12 (48%) consisted of a single-loop re-entry. Re-entry was located entirely in the LA in 24 (96%) and in the RA in 1 AFL (4%). Average TCL was 261 ± 35 ms. Activation maps contained an average 18,522 points (range 8216–33,368).

**Figure 1:**
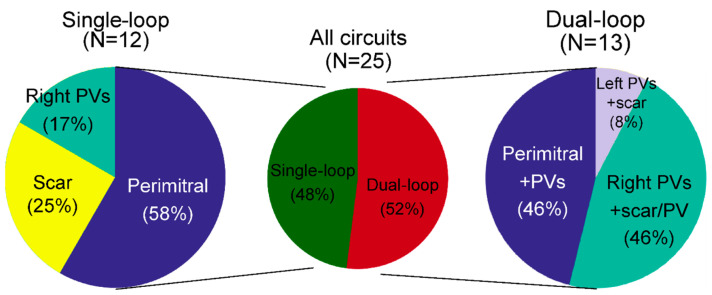
Circuit anatomy in single- and dual-loop macro-re-entrant atrial tachycardias, identified by the central obstacle defining each re-entrant loop. PV denotes pulmonary vein.

Table 2 and Figure 1 summarize the anatomy of single- and dual-loop AFLs. Single-loop circuits (N = 12) were distributed as follows: 6 perimitral (50% of single-loop AFLs), 2 around the right PVs, 2 coronary sinus (CS)-dependent flutters with an endo-epicardial component, 1 around a scar on the interatrial septum, and 1 around a scar on the RA free wall. Dual-loop circuits (N = 13) had the following paths: 4 perimitral with a second loop around the left PVs, 4 around the right PVs with a second loop around a line of conduction block, 2 perimitral with a second loop around the right PVs, 1 with a loop around each right PV, 1 around the right PVs with a second loop around the left PVs, and 1 around the left PVs with a second loop around a line of conduction block. The most common configuration of dual-loop re-entry was therefore a perimitral loop with a second roof-dependent loop (around either the right of left PVs), which accounted for 6 of 13 (46%) dual-loop AFLs.

**Figure 2:**
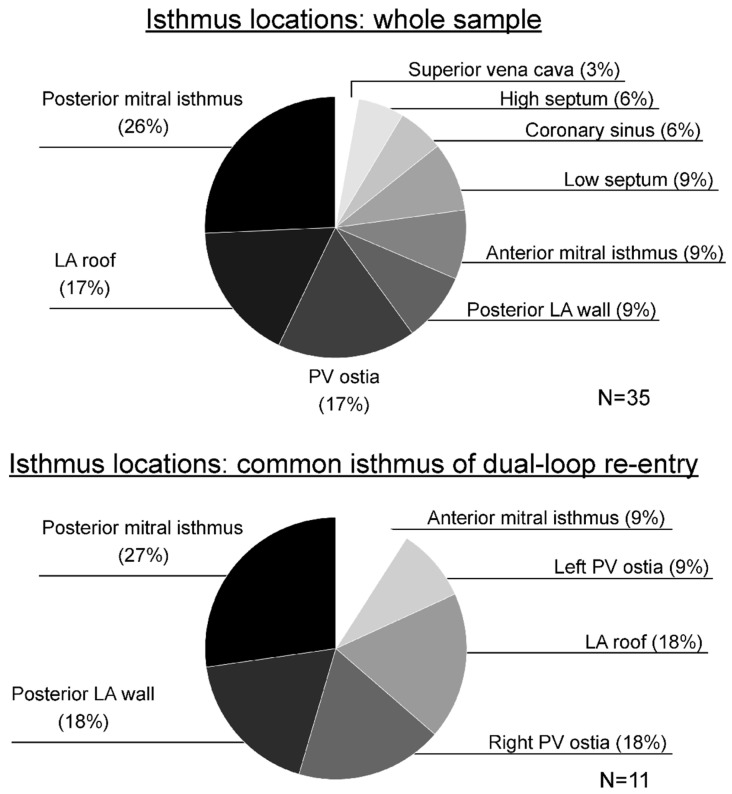
Location of narrow isthmuses (<30 mm). Top panel includes all identified narrow isthmuses in single- and dual-loop atypical atrial flutters. Bottom panel includes only narrow isthmuses located on the segment common to both loops in dual-loop re-entry. LA denotes left atrium; PV, pulmonary vein.

Isthmuses < 30 mm (not limited to the common isthmus in case of dual-loop re-entry) were identified at the following locations, and summarized in Table 3 and Figure 2: 9 at the posterior mitral isthmus (defined as the region between the left inferior PV and the posterior mitral annulus), 6 at the LA roof, 5 at a right PV ostium or carina, 3 at the posterior LA wall, 3 at the anterior mitral isthmus (defined as the region between the left superior PV/left atrial appendage and the anterior mitral annulus), 3 at the interatrial septum near the mitral annulus, 2 within the CS (epicardial), 2 at the interatrial septum near the right PVs, 1 at a left PV ostium, and 1 at the superior vena cava-RA free wall junction.

Compared to single-loop re-entry, dual-loop AFLs exhibited isthmuses characterized by greater isthmus width (17.1 ± 5.9 versus 11.4 ± 2.8 mm in dual-loop versus single-loop, respectively, *p* = 0.001), faster local conduction velocity (0.56 ± 0.42 versus 0.29 ± 0.16 m/s, respectively, *p* = 0.014), and greater bipolar electrogram voltage (0.53 ± 0.40 versus 0.27 ± 0.26 mV, respectively, *p* = 0.0499). PPI-TCL at isthmuses of dual- versus single-loop AFLs did not differ significantly (−6.6 ± 10.0 versus −12.8 ± 14.2 ms, respectively, *p* = 0.29). TCL did not differ significantly between dual- and single-loop AFLs (250 ± 28 versus 271 ± 38 ms, respectively, *p* = 0.14).

Among dual-loop AFLs, 11 (85%) exhibited a common isthmus < 30 mm along the common path of both re-entry loops, the location of which were as follows: 3 at the posterior mitral isthmus, 2 at the LA roof, 2 at a right PV ostium, 2 at the posterior LA wall, 1 at the anterior mitral isthmus, and 1 at a left PV ostium.

Among dual-loop AFLs, common isthmuses (N = 11) and secondary isthmuses (N = 12) did not differ significantly with respect to isthmus width (15.8 ± 5.9 versus 18.3 ± 5.7 mm, respectively, *p* = 0.33), local conduction velocity (0.45 ± 0.33 versus 0.68 ± 0.47 m/s, respectively, *p* = 0.23), bipolar electrogram voltage (0.52 ± 0.47 versus 0.55 ± 0.32 mV, respectively, *p* = 0.88), or local PPI-TCL (−11.1 ± 6.3 versus −2.6 ± 10.9 ms, respectively, *p* = 0.08).

### 3.3. Effect of Ablation

**Figure 3:**
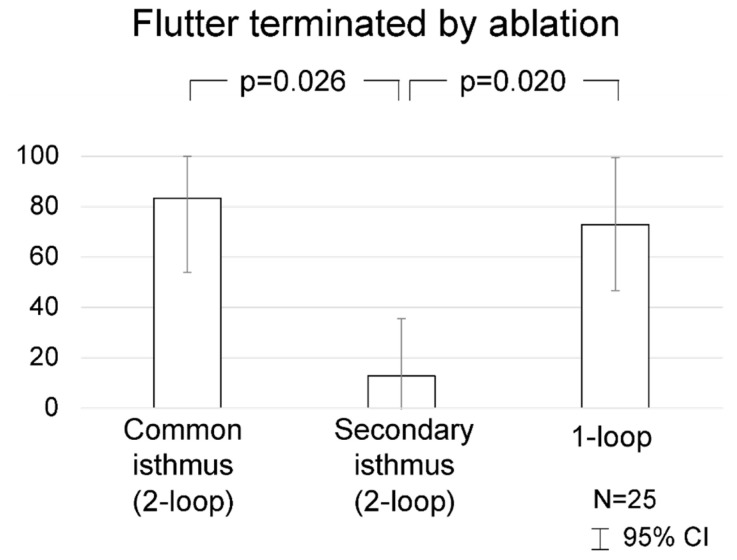
Rate of arrhythmia termination to sinus rhythm during radiofrequency ablation, by flutter type (single- or dual-loop) and isthmus type (common path or side loop).

In dual-loop AFLs, ablation more frequently resulted in AFL termination to sinus rhythm when targeting a common isthmus compared to a secondary isthmus (Figure 3): 5 of 6 (83%) common isthmus ablations versus 1 of 8 (13%) secondary isthmus ablations resulted in arrhythmia termination to sinus rhythm, *p* = 0.026. Ablation of a secondary isthmus resulted in sustained TCL increase > 15 ms in 5 of 8 (71%) and in a change in activation sequence in 2 of 8 (29%).

Ablation of the narrowest accessible isthmus of single-loop AFLs resulted in termination to sinus rhythm in 8 of 11 (73%), a rate similar to that of dual-loop common isthmus ablation (83%, *p* = 1.0) and higher than that of dual-loop secondary isthmus ablation (13%, *p* = 0.020).

### 3.4. Procedure Outcome

When accounting for all arrhythmias targeted for ablation during the 20 procedures (including those that were not fully mapped), ablation targets/lesions were: a posterior mitral line in 8, a pulmonary vein ostium or carina in 6, an LA roof line in 5, the interatrial septum in 3, an anterolateral mitral line in 3, the CS in 2, an anteroseptal line in 2 and the superior vena cava-RA free wall junction in 1. Procedure endpoint was tachycardia termination to sinus rhythm followed by non-inducibility in 14 of 20 (70%), ablation in sinus rhythm followed by non-inducibility in 2 of 20 (10%), transformation of AFL into AF during ablation followed by non-inducibility in 2 of 20 (10%) and some residual inducible sustained AFL in 2 of 20 (10%) which were not ablated due to multiple inducible atrial tachycardias that could not be readily mapped and/or ablated. Complete block across ablation lines was confirmed in 11 of 20 (55%) procedures, including 4 of 8 posterior mitral lines, 5 of 5 LA roof lines, 2 of 3 anterolateral mitral lines, 2 of 2 anteroseptal mitral lines and 1 of 1 superior vena cava-RA free wall line.

**Figure 4:**
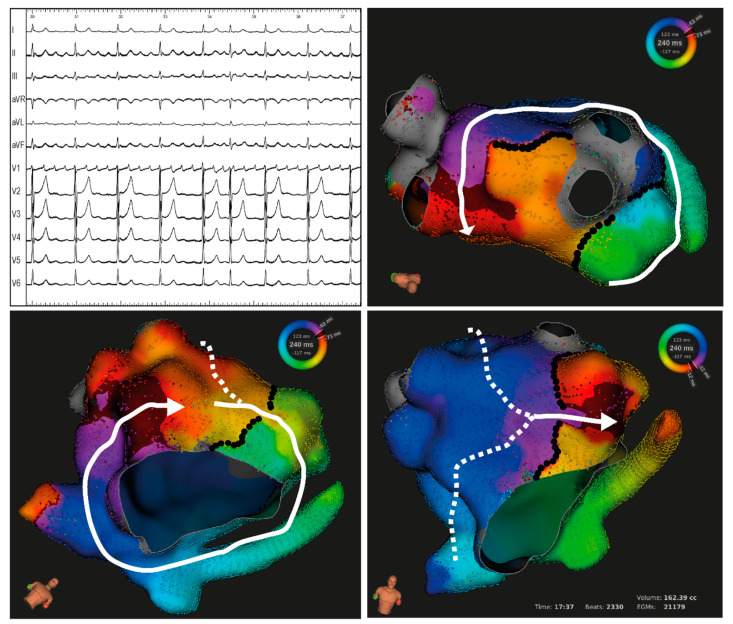
Example of a dual-loop atypical atrial flutter in a patient with a history of atrial fibrillation ablation. Top left panel shows 12-lead ECG with predominantly positive F waves in V1 and inferior leads. Top right and bottom panels show the left atrial activation map, depicting a dual-loop re-entry with one perimitral loop and a second roof-dependent loop around the left pulmonary veins. A narrow common isthmus is present at the anterior mitral isthmus. A narrow secondary isthmus is present at the left atrial roof. White arrows indicate the path of the leading wavefront. Black markers indicate existing lines of conduction block. Ablation was first targeted at the roof isthmus, resulting in slight cycle length prolongation (from 240 to 254 ms). The anterior mitral isthmus was then ablated, resulting in termination to sinus rhythm. Complete conduction block across both lines was then confirmed.

Follow-up data was available for 13 patients with a median follow-up of 429 (IQR 114–461) days. Of those, 5 (38%) exhibited arrhythmia recurrence after a median of 142 (IQR 114–429) days. Three of the patients with arrhythmia recurrence had exhibited at least one dual-loop AFL at the index procedure. Two patients with arrhythmia recurrence underwent repeat procedures, which showed recurrence of perimitral AFL in both cases: one patient previously had dual-loop perimitral and roof-dependent AFL (activation map of index procedure is shown in Figure 4) which was ablated by a roof line and anterolateral mitral line (with confirmed conduction block) and exhibited recurrence in the form of single-loop perimitral AFL due to conduction recovery across the anterolateral mitral line through an epicardial CS connection; the second patient previously had single-loop perimitral AFL which was ablated by a posterior mitral line (requiring CS lesions, and with confirmed conduction block) and exhibited recurrence in the form of single-loop perimitral AFL due to conduction recovery across the posterior mitral line.

## 4. Discussion

Dual-loop re-entry was the underlying mechanism in more than half of AFLs. The most common circuit path involved a perimitral loop with a second roof-dependent loop. Compared to single-loop re-entry, dual-loop re-entry was characterized by isthmuses exhibiting greater minimum width, faster local conduction velocity, and higher bipolar electrogram voltage. In case of dual-loop re-entry, ablation more frequently resulted in termination to sinus rhythm when targeting an isthmus on the segment common to both loops, with a termination rate similar to that observed in single-loop AFL ablation. In contrast, ablation of a secondary isthmus more frequently resulted in TCL increase or modification of the atrial activation sequence without arrhythmia termination to sinus rhythm.

### 4.1. Characteristics of Single- and Dual-Loop Atypical Atrial Flutter

Our results show a higher rate of dual-loop AFL (52% of AFLs in 63% of patients) compared to previous studies. In the largest study to specifically investigate multiple-loop macro-re-entrant atrial tachycardias, Takigawa et al. [1] identified 42 dual-loop and 2 triple-loop re-entries in 41 of 193 (21%) consecutive patients undergoing catheter ablation of post-AF ablation atrial tachycardia. In another study on post-AF ablation atrial tachycardia, Chae et al. [3] reported multiple-loop re-entry in 17 of 78 (22%) patients. Likewise, Derval et al. [2] studied 132 patients undergoing catheter ablation of atrial tachycardia using high-density electroanatomical mapping. They identified 27 dual-loop AFLs in 24 (18%) patients. The lower prevalence of dual-loop re-entry in those previous cohorts might be partly explained by the inclusion, in all three samples, of all patients with atrial tachycardia scheduled for ablation with a high-density mapping system. While patients with clinical presentation as CTI-dependent atrial flutter were excluded, a proportion of patients were eventually diagnosed with CTI-dependent atrial flutter as well as non-re-entrant atrial tachycardias. In contrast, these cases were not included in our series. Small sample sizes may also explain some variability in the prevalence of dual-loop AFL.

While the precise circuit path is highly individual, we found that variants of a perimitral loop with a second roof-dependent loop was the most common dual-loop configuration, representing 46% of dual-loop AFLs. Consistent with our findings, Takigawa et al. [1] found this configuration in 19 of 44 (43%) multiple-loop AFLs. Among the remaining circuits, the PVs/carinas were involved in most cases. Likewise, Derval et al. [2] reported that 22 of 27 (81%) dual-loop re-entries were perimitral with a second roof-dependent loop, 4 (15%) were perimitral with a second localized loop, and 1 (4%) was roof-dependent with a second localized loop. In the study by Chae et al. [3], the precise anatomy was not described for all dual-loop circuits, but the mitral isthmus was reported as the most common isthmus location.

The circuit path of dual-loop AFLs tends to differ based on underlying etiology. The majority of AFLs occur following catheter ablation of AF [2,8], in which case re-entry usually takes place in the LA [1,2,3], with the most common dual-loop anatomy being a combination of perimitral and roof-dependent re-entry, as discussed above. In the remaining cases, combinations of PV ostia and/or acquired lines of block were most commonly involved. Indeed, the existence of multiple anatomical obstacles and orifices in the LA (mitral annulus, PVs, and left atrial appendage) constitutes a baseline anatomical substrate propitious to the development of re-entry. Acquired conduction blocks from ablation scars, especially when in the vicinity of anatomical obstacles, promote re-entrant tachycardias by forming protected channels/isthmuses delimited on one side by an anatomical obstacle and on the other by the acquired scar. Protected channels enable re-entry by preventing re-entrant wavefronts from colliding laterally with their tail. Such channels commonly involve dual-loop re-entry, with each loop revolving around one of the channel-delimiting obstacles (e.g., mitral annulus and circumferential PV isolation lesions). As a result, the majority of post-AF ablation dual-loop AFLs involve one or two anatomical obstacles, as shown in the present analysis (Table 2) as well as previous series [1,2]. Dual-loop atrial re-entry in patients with a history of cardiac surgery have been predominantly reported as RA re-entry with one CTI-dependent loop and a second peri-atriotomy loop [4,5]. As in post-AF ablation AFL, the predominant substrate of dual-loop re-entry therefore consisted of a iatrogenic block in the vicinity of an anatomical obstacle (the tricuspid annulus). AFL in the absence of iatrogenic atrial scars is rarer and has been infrequently studied. In one study on 223 patients undergoing catheter ablation of left atrial tachycardia, Schaeffer et al. identified 15 (6.7%) patients with LA re-entry and no prior LA-modifying cardiac interventions [6]. The authors report a typical phenotype involving a scar on the anterior LA wall which facilitated re-entry, most commonly in the form of perimitral AFL. In that study, 7 of 15 (47%) patients with “idiopathic” AFL exhibited dual-loop re-entry, all of which consisted of a perimitral loop with a second loop around a scar on the anterior LA. Again, the common underlying substrate was a protected channel formed by an anatomical obstacle and a nearby acquired conduction block. In the study by Derval et al. [2] mentioned above, 16 of 132 (12%) patients had no prior history of cardiac intervention. These patients accounted for 3 of 24 (13%) patients with dual-loop re-entrant atrial tachycardias; detailed anatomy was not reported for this subgroup.

In the present study, isthmuses of dual-loop AFLs exhibited greater minimum width, faster conduction velocity, and higher bipolar electrogram voltage compared to single-loop AFLs. While our sample size is too small for outcome comparisons, ablation targets exhibiting such electrophysiological characteristics have been associated with poorer long-term rhythm outcomes [1,2]. These characteristics may be markers of less vulnerable conduction and therefore less readily ablated targets, as opposed to narrow slow-conducting low-voltage isthmuses [7]. The present findings therefore suggest that, in addition to the anatomical complexity, dual-loop AFLs may be more challenging to ablate due to unfavorable characteristics of potential target isthmuses.

### 4.2. Implications for Mapping and Catheter Ablation

The present data shows dual-loop re-entry as the most common underlying mechanism of AFL after analysis of complete high-density electroanatomical maps. The prevalence of dual-loop re-entry could be underestimated in clinical practice, since failure to identify dual-loop re-entry may occur despite an apparently complete activation map: documentation of the entire TCL can be achieved by mapping only one of the two re-entry loops. In addition, adequate entrainment mapping criteria with PPI-TCL ≈ 0 ms can be obtained along both loops: average PPI-TCL on secondary isthmuses was −2.6 ± 10.9 ms in the present study. Dual-loop re-entry should therefore be kept in mind as a potential pitfall in AFL mapping. The best strategy to avoid this pitfall may be the systematic acquisition and careful analysis of anatomically complete high-density activation maps.

Given the higher acute efficacy of ablation when targeting the common segment of dual-loop AFLs, as opposed to targeting a secondary isthmus along a side loop, our results suggest that systematic identification of dual-loop re-entry and adequate choice of ablation targets should lead to more selective and efficient ablation strategies. In contrast, incorrect classification of one of two loops as a single-loop re-entry may result in ineffective lesion creation and/or tachycardia modification without termination. Indeed, ablation of a secondary isthmus more frequently resulted in TCL increase or activation sequence modification without tachycardia termination, most commonly due to continuation of the arrhythmia along the second unablated re-entry loop (as illustrated in Figure 4). Most importantly, failure to identify dual-loop re-entry may lead to incomplete elimination of the arrhythmia substrate. Indeed, ablation strategies should take into account the risk of arrhythmia recurrence based on arrhythmia mechanism and individual anatomy. In case of dual-loop re-entry, it appears reasonable to consider anatomically complete elimination of all re-entry loops to optimize long-term rhythm outcomes, e.g., ablation of both the common isthmus and a secondary isthmus or ablation of two secondary isthmuses [5]. In addition, to achieve durable results, lesion sets should consist of complete lines of block made of anatomically contiguous lesions and anchored to anatomical (or electrically silent) obstacles. It should also be noted that perimitral re-entry, which was present in most dual-loop AFLs, has been reported to exhibit a high rate of recurrence after ablation [9]. Complete block along mitral lines can be challenging to obtain and often require epicardial ablations within the CS. Likewise, reconnection has been reported to involve epicardial connections in the majority of recurrences [9].

Recently, Santucci et al. [10] proposed a framework to conceptually unify dual- and single-loop re-entry based on the observation that two distinct critical boundaries (obstacles that border ≥ 90% of TCL) could be identified in all cases of macro-re-entrant atrial tachycardias. From that perspective, when the two boundaries are close to each other, the re-entry mechanism is traditionally classified as dual-loop re-entry, and when the two boundaries are on opposite sides of the chamber, the circuit is traditionally classified as single-loop re-entry. The authors tested an ablation strategy based on creating lesions that connect the two critical boundaries (directly or indirectly via non-critical boundaries) and reported arrhythmia termination in 123 (96%) of 128 cases (including single-loop reentry). While the framework is essentially conceptual in nature, these results highlight the benefit of obtaining complete high-density activation maps of the entire chamber of interest, and of pursing an ablation strategy based on detailed assessment of the complete re-entry circuit.

While the prevention of iatrogenic AFL is beyond the scope of the present study, some insight can be gained from our findings. Given that dual-loop AFL frequently arises in the setting of ablation scars close to anatomical obstacles, one may hypothesize that anchoring ablation lesions to nearby anatomical obstacles could decrease pro-arrhythmia. Likewise, optimizing for durable lines of block through the achievement of irreversible, transmural, anatomically contiguous lesions, may help prevent iatrogenic AFL. However, the effects of such strategies on long-term rhythm outcomes have yet to be determined prospectively.

Given the high prevalence of perimitral re-entry in post-AF ablation AFL (often with a second PV-dependent or roof-dependent loop), mitral isthmus ablation lines, among other lesion sets, have been proposed as an adjunct to PV isolation in AF ablation. Of note, systematic ablation lines (including at the mitral isthmus) at de novo AF ablation have failed to improve overall rhythm outcomes in past randomized trials [11]. Other strategies are currently being evaluated prospectively [12]. Pending further data, our current practical approach to de novo AF ablation involves the individualized assessment of arrhythmia inducibility by atrial burst pacing after PV isolation (primarily to assess AF inducibility and to guide anti-AF substrate modification), and thereafter in a stepwise fashion following AF substrate modification. In the presence of induced sustained AFL, AFL is mapped and targeted for ablation, taking into account AFL anatomy and individual factors such as age and AF type (paroxysmal or persistent).

### 4.3. Limitations

The main limitation of the present study is its small sample size. In addition, given the observational design, any inference regarding ablation endpoints and implications for ablation strategies should be interpreted with the usual caution. Long-term outcome data is reported for descriptive purposes but cannot be used for meaningful statistical analysis. It should also be noted that the respective value of ablation strategies targeting two isthmuses versus one common isthmus have not been established and remain to be evaluated prospectively.

General technical limitations of electroanatomical mapping systems apply to our study. For example, epicardial activation cannot be mapped endocardially, which may result in incomplete or misleading activation maps. Moreover, automated local activation time annotation, cardiac and respiratory movement filters, and catheter stability filters, are all subject to errors that may lead to inaccuracies.

## 5. Conclusions

Dual-loop re-entry was the underlying mechanism in more than half of macro-re-entrant atrial tachycardias in patients undergoing catheter ablation of AFL. The most common circuit path involved a perimitral loop with a second roof-dependent loop. Compared to single-loop re-entry, dual-loop re-entry was characterized by isthmuses of greater minimum width, faster conduction velocity, and higher bipolar electrogram voltage. Ablation more frequently resulted in tachycardia termination when targeting an isthmus common to both loops, with a termination rate similar to single-loop AFL ablation. In contrast, ablation of a secondary isthmus on a side loop more frequently resulted in TCL increase or activation sequence modification without arrhythmia termination. Given the high prevalence of dual-loop re-entry and the associated pitfalls, careful assessment of complete activation maps should be included in a systematic approach to AFL mapping. Ablation strategies should consider complete elimination of all re-entrant loops.

## Figures and Tables

**Table 1:** Baseline characteristics.

	All Patients (N = 19)
Age (years)	66 (±15)
Male gender	15 (79%)
BMI (kg/m^2^)	25.9 (±4.6)
Associated conditions	
Arterial hypertension	8 (42%)
Diabetes mellitus	1 (5.3%)
Dyslipidemia	5 (26%)
Structural heart disease	5 (26%)
Prior stroke or transient ischemic attack	3 (15.8%)
CHA_2_DS_2_-VASc score	
0–1	8 (42%)
≥2	11 (58%)
LA surface (cm^2^)	24.9 (±5.1)
RA surface (cm^2^)	20.3 (±5.2)
LVEF ≤ 50%	4 (21%)

Data presented as mean (±standard deviation) for continuous variables and number (percentage) for categorical variables. BMI denotes body mass index; LA, left atrium; LVEF, left ventricular ejection fraction; RA, right atrium.

**Table 2:** Anatomy of single- and dual-loop atypical atrial flutters.

	Single-Loop Re-Entry (N = 12)	Dual-Loop Re-Entry (N = 13)	
Perimitral	6 (50%)	6 (46%)	4 with second loop around left PVs
			2 with second loop around right PVs
Around right PVs	2 (17%)	6 (46%)	4 with second loop around a line of block
			1 with one loop around each right PV
			1 with second loop around left PVs
Coronary sinus-dependent	2 (17%)		
Around a septal scar	1 (8%)		
Around a right atrial free wall scar	1 (8%)		
Around left PVs		1 (8%)	Second loop around a functional block

Data described as number (percentage). PV denotes pulmonary vein.

**Table 3:** Anatomical location of isthmuses.

	All Isthmuses	Common Isthmuses of Dual-Loop Reentries
Posterior mitral isthmus	9 (26%)	3 (27%)
Pulmonary vein ostium	6 (17%)	3 (27%)
Left atrial roof	6 (17%)	2 (18%)
Posterior left atrial wall	3 (9%)	2 (18%)
Anterior mitral isthmus	3 (9%)	1 (9%)
Inferior interatrial septum	3 (9%)	
Coronary sinus (epicardial)	2 (6%)	
Superior interatrial septum	2 (6%)	
Superior vena cava—right atrial free wall junction	1 (3%)	

Data described as number (percentage). PV denotes pulmonary vein.

## Data Availability

The original data is not readily available due to privacy concerns.

## References

[1] Takigawa M., Derval N., Maury P., Martin R., Denis A., Miyazaki S., Yamashita S., Frontera A., Vlachos K., Kitamura T. (2018). Comprehensive Multicenter Study of the Common Isthmus in Post-Atrial Fibrillation Ablation Multiple-Loop Atrial Tachycardia. Circ. Arrhythmia Electrophysiol..

[2] Derval N., Takigawa M., Frontera A., Mahida S., Vlachos K., Denis A., Duchateau J., Pillois X., Yamashita S., Berte B. (2020). Characterization of Complex Atrial Tachycardia in Patients With Previous Atrial Interventions Using High-Resolution Mapping. JACC Clin. Electrophysiol..

[3] Chae S., Oral H., Good E., Dey S., Wimmer A., Crawford T., Wells D., Sarrazin J.-F., Chalfoun N., Kuhne M. (2007). Atrial Tachycardia After Circumferential Pulmonary Vein Ablation of Atrial Fibrillation: Mechanistic Insights, Results of Catheter Ablation, and Risk Factors for Recurrence. J. Am. Coll. Cardiol..

[4] Seiler J., Schmid D.K., Irtel T.A., Tanner H., Rotter M., Schwick N., Delacrétaz E. (2007). Dual-loop circuits in postoperative atrial macro re-entrant tachycardias. Heart.

[5] Shah D., Jaïs P., Takahashi A., Hocini M., Peng J.T., Clementy J., Haïssaguerre M. (2000). Dual-Loop Intra-Atrial Reentry in Humans. Circulation.

[6] Schaeffer B., Akbulak R.Ö., Jularic M., Moser J., Eickholt C., Schwarzl J.M., Klatt N., Kuklik P., Meyer C., Willems S. (2019). High-Density Mapping and Ablation of Primary Nonfocal Left Atrial Tachycardia: Characterizing a Distinct Arrhythmogenic Substrate. JACC Clin. Electrophysiol..

[7] Johner N., Shah D.C., Jousset F., Dall’Aglio P.B., Namdar M. (2019). Electrophysiological and Anatomical Correlates of Sites With Postpacing Intervals Shorter Than Tachycardia Cycle Length in Atypical Atrial Flutter. Circ. Arrhythmia Electrophysiol..

[8] Laţcu D.G., Bun S.-S., Viera F., Delassi T., El Jamili M., Al Amoura A., Saoudi N. (2017). Selection of Critical Isthmus in Scar-Related Atrial Tachycardia Using a New Automated Ultrahigh Resolution Mapping System. Circ. Arrhythmia Electrophysiol..

[9] Takigawa M., Derval N., Martin C.A., Vlachos K., Denis A., Nakatani Y., Kitamura T., Cheniti G., Bourier F., Lam A. (2020). Mechanism of Recurrence of Atrial Tachycardia: Comparison Between First Versus Redo Procedures in a High-Resolution Mapping System. Circ. Arrhythmia Electrophysiol..

[10] Santucci P.A., Bhirud A., Vasaiwala S.C., Wilber D.J., Green A. (2024). Identification of 2 Distinct Boundaries Distinguishes Critical From Noncritical Isthmuses in Ablating Atypical Atrial Flutter. JACC Clin. Electrophysiol..

[11] Verma A., Jiang C., Betts T.R., Chen J., Deisenhofer I., Mantovan R., Macle L., Morillo C.A., Haverkamp W., Weerasooriya R. (2015). Approaches to catheter ablation for persistent atrial fibrillation. N. Engl. J. Med..

[12] Pambrun T., Denis A., Duchateau J., Sacher F., Hocini M., Jaïs P., Haïssaguerre M., Derval N. (2019). MARSHALL bundles elimination, Pulmonary veins isolation and Lines completion for ANatomical ablation of persistent atrial fibrillation: MARSHALL-PLAN case series. J. Cardiovasc. Electrophysiol..

